# The Haemodynamic Response to Endotracheal Intubation at Different Time of Fentanyl Given During Induction: A Randomised Controlled Trial

**DOI:** 10.1038/s41598-020-65711-9

**Published:** 2020-06-01

**Authors:** Cheng Yeon Teong, Chien-Chung Huang, Fang-Ju Sun

**Affiliations:** 1 Department of Anesthesiology, MacKay Memorial Hospital, Taipei, Taiwan, ROC; 2 MacKay Medical College, New Taipei City, Taiwan, ROC; 3 MacKay Junior College of Medicine, Nursing and Management College, Taipei, Taiwan, ROC; 4 Department of Medical Research, MacKay Memorial Hospital, Taipei, Taiwan, ROC

**Keywords:** Drug discovery, Physiology, Health care, Drug regulation, Medical research, Drug development, Clinical trial design

## Abstract

Endotracheal intubation elicits huge spectrum of stress responses which are hazardous in high-risk patients. Numerous drugs and techniques have been applied to attenuate the stress responses. In this double-blind study, one hundred and forty-five patients over 20 years old, ASA physical status I and II, undergoing elective surgeries requiring general anaesthesia with endotracheal intubation were included. Patients were randomly divided into three groups which fentanyl 2 mcg/kg was given at either 1, 2, 3 minutes before intubation. All groups received midazolam 0.05 mg/kg, lidocaine 0.5 mg/kg, propofol 2 mg/kg and rocuronium 1 mg/kg before intubation. Haemodynamic parameters were recorded for 10 minutes after induction. Two-level longitudinal hierarchical linear models were used for data interpretation and P < 0.05 was considered statistically significant. The study demonstrated significantly lower haemodynamic responses in the group who received fentanyl 2 minutes before intubation (P < 0.05). Confounding factors such as smoking, hypertension, diabetes mellitus and preoperative intravenous fluid supplement were analysed. In conclusion, fentanyl injection 2 minutes before intubation is recommended in order to obtain the most stable haemodynamic status.

## Introduction

Laryngoscopy and intubation induce huge spectrum of stress responses such as tachycardia and hypertension. Those are in association with the surge of plasma adrenaline concentration following intubation^1^. A sudden change in haemodynamic status may precipitate myocardial ischemia, especially in high-risk patients. Therefore, many approaches have been introduced to attenuate the stress response^2^.

Fentanyl, a fast-acting synthetic μ receptor-stimulating opioid, has been commonly prescribed in preventing the sympathetic stimulation during intubation^3–5^. As summarisation from the previous knowledge, the dosage for intubation is 1–3 mcg/kg and it can last for 30–60 minutes^6–8^. There are few studies discussing the optimal dosage for intubation in order to minimise the changes on systemic haemodynamics^3,4,9^.

Nonetheless, there is scarce study depicting the haemodynamic responses in relation to the time of fentanyl given before intubation^10^. Therefore, we conduct a prospective, double-blind, randomised-controlled clinical trial to examine the optimal timing for fentanyl administration to maintain stable vital signs throughout induction.

## Results

Of the 145 patients enrolled, there are no patient characteristics and demographic differences among groups (Table 1) and all were intubated once by laryngoscope within 30 seconds without complications and difficulties. The data were completely obtained and divided into all time (Table 2), before intubation (time 0–3) and after intubation (time 4–10) (Table 3) for statistical analyses.

**Table 1:** Comparison of patient characteristics in three groups.

	F3	F2	F1	*P*
Patients	49	48	48	
Age (yr), mean (SD)	41 (11)	43 (11)	44 (9)	0.446
Sex (F/M)	31/18	27/21	28/20	0.770
Weight (kg), mean (SD)	64.6 (12.2)	63.9 (12.2)	60.9 (11.2)	0.266
Height (cm), mean (SD)	163 (9)	164 (8)	163 (8)	0.723
SBP0 (mmHg), mean (SD)	128.4 (20.1)	130.2 (18.8)	125.3(16.8)	0.420
DBP0 (mmHg), mean (SD)	77.1 (11.5).	77.6 (12.0)	75.9 (12.0)	0.781
HR0 (/min), mean (SD)	80.1 (13.8)	79.2 (14.9)	79.4 (15.2)	0.901
ASA I/II	10/39	9/39	7/41	0.744
Smoking, n (%)	9 (18.4)	6 (12.5)	8 (16.7)	0.719
HTN, n (%)	8 (16.3)	6 (12.5)	7 (14.6)	0.866
DM, n (%)	2 (4.1)	6 (12.5)	1 (2.1)	0.080
IVF, n (%)	24 (49.0)	31 (64.6)	27 (56.3)	0.300

(SBP0 = baseline systolic blood pressure; DBP0 = baseline diastolic blood pressure; HR0 = baseline heart rate; HTN = hypertension; DM = Diabetes mellitus; IVF = preoperative intravenous fluid supplement)

Age, weight, height, SBP, DBP and HR are expressed as means. There was no significant difference among groups.

**Table 2:** Comparison of data among three groups in relevant to SBP and HR throughout all time.

	SBP				HR			
	B	95% CI		*P*	B	95% CI		*P*
Group F3^§^	−5.44	−7.57	−3.31	<0.001*	−2.76	−4.19	−1.32	<0.001*
Group F2^§^	−5.03	−7.18	−2.88	<0.001*	−3.42	−4.86	−1.98	<0.001*
Smoking	3.13	0.36	5.90	0.027*	5.29	3.41	7.17	<0.001*
HTN	3.80	0.58	7.02	0.021*	−2.04	−4.26	0.14	0.066
DM	−7.14	−11.52	−2.76	0.001*	−4.79	−7.75	−1.85	0.001*
IVF	−1.13	−3.27	1.01	0.302	−1.70	−3.17	−0.23	0.023*

(*P < 0.05 implies significant differences; ^§^compared to group F1).

**Table 3:** Comparison of data among groups in relevant to SBP and HR after intubation (time 4–10).

	SBP				HR			
	B	95% CI		*P*	B	95% CI		*P*
Group F3^§^	−3.48	−5.92	−1.04	0.005*	−2.02	−3.61	−0.42	0.013*
Group F2^§^	−4.39	−6.85	−1.92	0.001*	−3.93	−5.53	−2.32	<0.001*
Smoking	4.88	1.69	8.08	0.003*	7.51	5.41	9.62	<0.001*
HTN	3.84	0.14	7.55	0.042*	−2.49	−4.93	−0.05	0.046*
DM	−7.01	−12.07	−1.96	0.007*	−5.51	−8.42	−1.80	0.003*
IVF	−1.51	−3.97	0.95	0.228	−2.32	−3.97	−0.67	0.006*

(*P < 0.05 implies significant differences; ^§^compared to group F1)

Haemodynamic differences were demonstrated and the trend is synonymous with Table 2 (all time)

*All time (*Table 2*)*. SBP and HR were relatively lower in group F3 (P < 0.001) and F2 (P < 0.001) compared with group F1 throughout the induction course. There were also statistically significant increases in SBP (P = 0.027) and HR (P < 0.001) in smoking patients. Nevertheless, patients with hypertension presented with higher SBP (P = 0.021) while DM patients have decreases of SBP and HR. Patients with preoperative intravenous fluid (IVF) supplement have a lower HR among groups (P = 0.023).

Group F2 and F3 have lower SBP and HR compared to group F1. Patients with histories of smoking, hypertension, DM and preoperative IVF supplement have significant differences on haemodynamics.

*Before intubation (Time 0–3) (table not shown)*. SBP was significantly lower in group F3 (B = −10.13, P < 0.001) and F2 (B = −6.67, P = 0.001) compared with group F1 before intubation. Yet, only lower HR was noted in group F3 when comparing to group F1 (B = −4.34, P < 0.001). HR in DM patients decreased at this time (P = 0.03). However, there was no significant difference among groups in smoking, HTN and preoperative IVF supplement.

*After intubation (Time 4–10) (*Table 3*)*. After intubation, lower haemodynamic parameters were revealed in group F3 (P = 0.005) and F2 (P = 0.001). The smoking population has both higher SBP and HR. An increase of SBP and a decrease of HR were also noted in the patients with hypertension. Patients with preoperative IVF supplement have a lower HR among groups (P = 0.006).

### Summary

The trend of haemodynamic changes throughout the time is displayed in Fig. 3, which demonstrated a sudden surge of SBP and HR at the time of intubation and following by a drop. This is especially seen in group F1 that revealed drastic changes after intubation. SBP and DBP (data not shown) were lower in group F3 and F2 compared to group F1 before and after intubation. Group F3 had lower BP before intubation (B: −10.13 vs. −6.67) and higher BP after intubation (B: −3.48 vs. −4.39) than F2 when comparing with group F1. Thus, group F2 had less fluctuation of BP than F3.

**Figure 1:**
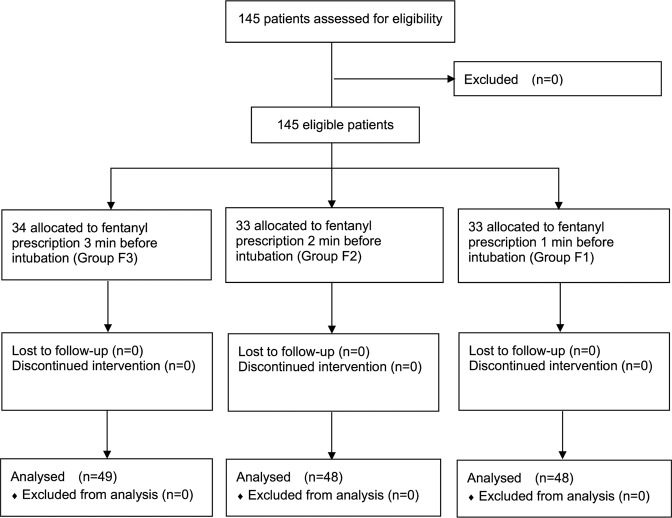
Consort Diagram.

**Figure 2:**
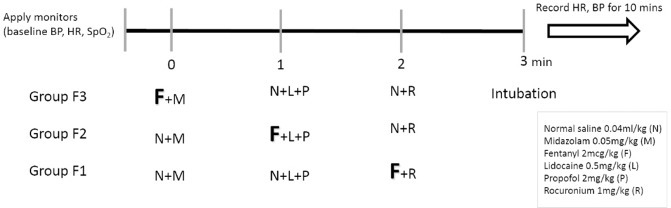
Flow chart of study protocol.

**Figure 3:**
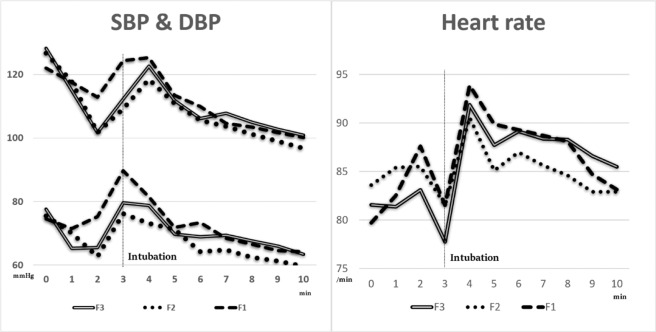
Comparison of haemodynamic changes among groups. Sudden surge of SBP, DBP and HR at the time of intubation following by a drop.

When mentioned HR, group F2 had comparable HR with F1 (P = 0.08) but group F3 had lower HR than group F1 (B: −4.34, P < 0.001) before intubation. Higher HR after intubation was found in group F3 (B: −2.02 vs. −3.93) than F2 when comparing with group F1. Therefore, group F2 had less fluctuation of HR than F3.

In brief, group F2, which represented the fentanyl injection 2 minutes before intubation, presented the most stable haemodynamics with least fluctuation throughout the induction course. Among the confounding factors being analysed, higher haemodynamic parameters in smoking population, lower haemodynamic parameters in DM patients and lower HR in patients with preoperative IVF supplement were observed.

## Discussion

Laryngoscopy and endotracheal intubation lead to the rush of adrenaline which causes the haemodynamic fluctuation^1,11,12^. The systemic haemodynamic changes do not place a threat to healthy patients but may increase the risks of morbidity and mortality in patients predisposed with coronary artery disease, recent myocardial infarction, hypertension, preeclampsia and cerebrovascular pathology^4,11^.

Therefore, various studies have been published regarding to the prevention of abrupt haemodynamic changes following endotracheal intubation^2–4,11,13–15^. To abate such condition, anaesthesiologists could apply several medications, such as beta-blockers, calcium channel blockers, lidocaine, propofol and opioids^4,11,13–17^. Aside from diverse medications, different intubating skills and methods can reduce haemodynamic changes such as fiberoptic intubation^12,18^, optimal fentanyl dosage^4,9,19^ and routes of drugs given^19^.

Yukari *et al*. examined the optimal dosage of fentanyl to diminish systemic haemodynamic swings during the induction. The researchers discovered that fentanyl 2 mcg/kg in patients without hypertension and 4 mcg/kg in those with hypertension are preferable in order to minimize the changes in vital signs and cardiac output associated with tracheal intubation^4^.

The onset time of fentanyl and its peak plasma concentration are dependent on the dosage employed and the means of delivery^3,9^. Immediate analgesic effect of fentanyl may occur as soon as 1 minute after intravenous injection^5^. KO *et al*. designed a research that patients received fentanyl (2 mcg/kg) 1, 3, 5, or 10 min before tracheal intubation and concluded that the optimal time of fentanyl injection is 5 minutes by comparing circulatory changes between baseline and 1 minute post-intubation^10^. The trend of haemodyamic fluctuation may not be precisely interpreted with intercomparing only 2 time points, particularly the gap between the drop before intubation and the rise after intubation. Patients could be harmed from the haemodyamic differences.

All groups presented a decrease of SBP, DBP and HR after induction and a sharp raise after intubation and followed by a fall. When speaking of group F1, higher haemodynamics and drastic changes might be partially responsible to the late onset of fentanyl in relative to the timing of intubation. On the other hand, group F3 with the early achievement of fentanyl peak effect leads to a lesser SBP, DBP and HR before intubation than group F2. The timing of fentanyl injection was altered without changing other parameters such as dosage and adjuvant medications, which hinted the importance of reaching the peak effect of fentanyl during intubation in order to lower the harmful stress responses.

Confounding factors were also analysed. Smoking population has higher haemodynamic parameters where the DM patients have lower haemodynamic parameters among groups. The effect is especially profound after intubation. Nicotine in tobacco is related to sympathetic nervous system overactivity^20,21^ and may cause arterial atherosclerosis which further narrowing the intravascular lumen^22,23^, leading to cardiovascular events eventually. It is also postulated that the cardiovascular autonomic neuropathy in DM patients^24^.

In addition, patients with preoperative IVF supplement have significantly lower HR throughout the procedure. It is anticipated that preoperative fluid optimization stabilizes HR in the concept of baroreceptor mediated reflex^25^. Abrupt surge of HR may lead to perioperative myocardial injury and should be avoided in specific population^26^.

There are limitations in this study. First, the accuracy of actual BP readings might be vary owing to the improper size of BP cuff^27^ and patient mobilisation since noninvasive blood pressure measurement was applied. Moreover, the exact BP value might be lost at the time of intubation due to the delay measurement of non-continuous noninvasive BP cuff. Thus, the top values of SBP and HR appeared at 4 minute in Fig. 3.

Second, patients with well-controlled hypertension were included. However, there was no record on the type of anti-hypertensive medications been taken and hence it may affect the magnitude of haemodynamic changes.

Third, preoperative IVF supplement has been analysed for one of the confounding factors. However, the discrepancy of fasting duration may affect the preoperative fluid status and further influence the haemodynamic parameters.

In conclusion, the timing of fentanyl administration is important during induction in order to obtain the most stable haemodynamic status. Higher haemodynamic parameters in smoking population, lower haemodynamic parameters in DM patients and lower HR in patients with preoperative IVF supplement were noted. Fentanyl injection 2 minutes before intubation is recommended.

## Methods

### Study design

The study was registered in the ClinicalTrials.gov Protocol Registration and Results System (PRS; registry number: NCT03728686; release date: 01/11/2018). After obtaining ethics committee approval (MacKay Memorial Hospital Institutional Review Board, protocol number: 16MMHIS097e; approval date: 16/12/2016), the protocol was done in accordance with relevant guidelines and all patients were informed consents. 145 patients with ASA physical status class I or II who undergoing elective, non-cardiac surgeries were recruited and randomly allocated by using computer-generated random number table into 3 groups (Fig. 1). Patients who were younger than 20 years old or with opioids allergy history were excluded from the study.

Fentanyl 2 mcg/kg was given at either 1, 2, 3 minutes before intubation which then allocated into group F3, F2 and F1 correspondingly. The random allocation sequence generation and group allocation were done by an anaesthesiologist who was not aware of the study protocol and was not participating in the study. Participants were enrolled by one investigator. The anaesthesiologist, who was in charge of intubation, was blinded from the patients’ group.

All patients were monitored and recorded for baseline vital signs including heart rate (HR), systolic blood pressure (SBP) and diastolic blood pressure (DBP) when arriving at the operating room. Midazolam 0.05 mg/kg, fentanyl 2 mcg/kg, lidocaine 0.5 mg/kg, propofol 2 mg/kg and rocuronium 1 mg/kg were administrated for induction of general anaesthesia according to the sequence as displayed in Fig. 2. Fully paralysed was obtained and endotracheal intubation with a maximum time of 30 seconds was attempted 3 minutes after injecting the induction agents. The patients’ vital signs were recorded every minute for 10 minutes after induction.

### Statistical analyses

The preliminary SBP results in baseline and the first intervention time point in all groups in the pilot study were 128.6 (SBP at baseline) and 100.1 (SBP at all induction medications given) (mean difference = 28.5) of group F3, 130.2 and 102.7 (mean difference = 27.5) of group F2, and 125.3 and 104.8 (mean difference = 20.5) of group F1. A repeated measures ANOVA was used to test hypotheses about means when there were two dependent factors (baseline and the first intervention in all groups) in the design. These dependent factors were termed within-subject factors as the same subjects were used for each level of the variable. Independent factors (three groups) could also be added to a repeated measures ANOVA design and were termed between-subject factors as different subjects were used for each level of the variable.

Then the effect size f that counted was 0.20 (medium effect), to achieve a power of greater than 80% with α value less than 0.05 (2-sided), and a sample size of 132 (44 in each group) was required for an adequately powered study (using software – G*Power 3.1). Considering the possibility of participants dropout during the study, total number of 145 patients were recruited^28^.

The data were evaluated by the two-level longitudinal hierarchical linear models (HLM) when being correlated between the time point t and its previous time point t-1 within a given patient, and to assess differences of SBP, DBP and HR among three groups over time. Restricted maximum likelihood estimation method (REML) was utilized. The longitudinal SBP, DBP or HR measurement (level 1) that was nested within patients (level 2). Because the model could assume random intercept (patients) and random slope (several time points) of measurement. Random effects accounted for within-patient correlation and were fitted a variance component (VC) covariance structure.

Finally, baseline variables were adjusted and entered as covariates (fixed effects) for potential confounding factors: gender, age, BMI, ASA, smoking, hypertension, diabetes mellitus (DM), intubating tools, preoperative intravenous fluid (IVF) supplement, and baseline value. Two-level HLM was used to compare mean change of SBP, DBP or HR over time and to detect the potential associations between these outcomes and main covariates. All reported P values were based on two-sided tests and were considered statistically significant if they were less than 0.05. Data were analyzed using IBM SPSS release 21.0 (IBM, Armonk, New York).
